# A novel 6-day cycle surgical pathology rotation improves resident satisfaction and maintains Accreditation Council for Graduate Medical Education (ACGME) milestone performance

**DOI:** 10.1016/j.acpath.2023.100088

**Published:** 2023-06-30

**Authors:** Christopher Felicelli, Alcino Pires Gama, Yevgen Chornenkyy, Kruti Maniar, Luis Z. Blanco, Jorge E. Novo

**Affiliations:** a Department of Pathology, Northwestern University, Feinberg School of Medicine, Chicago, IL, USA; b Department of Pathology and Laboratory Medicine, Northshore University, Evanston, IL, USA

**Keywords:** ACGME milestones, Quality improvement, Resident education, Surgical pathology

## Abstract

Surgical pathology residency training in the United States lags behind other specialties in quality control and graduated responsibility to train independent pathologists capable of seamlessly entering practice after training. We observed that our traditional 3-day-cycle surgical pathology cycle (day 1–grossing; day 2 –biopsies/frozens/preview; day 3 – sign-out) consistently and negatively impacted resident education by reducing preview time, case follow-up, immunohistochemical stain (IHC) interpretation, and molecular study integration. We aimed to create a modern surgical pathology rotation that improved performance and outcomes. We innovated our rotation to enhance resident education and ensure graduated responsibility. A novel 6-day cycle was created composed of 2 grossing days, 1 frozens/biopsies/preview days, 2 dedicated sign-out days, and 1 frozens/biopsies/case completion day. Residents completed surveys before implementing the new rotation and 6 months after implementation to track self-assessment of Accreditation Council for Graduate Medical Education (ACGME) milestone performance and internal quality control metrics. Clinical Competency Committee (CCC) annual evaluations were assessed in paired PGY levels pre- and post-intervention. After implementation, there was a statistically significant improvement in self-assessment of levels 4 and 5 of ACGME milestones and improved satisfaction of quality metrics, including time for previewing, reviewing IHC, graduated responsibility, and perceived readiness for independent practice. CCC evaluations showed overall maintained performance levels, with trends towards improvements in junior resident classes. Our 6-day cycle adequately fulfills the current demands of our sizeable academic center's surgical pathology training and can be a model for pathology residencies looking to modernize their surgical pathology rotations and resident education.

## Introduction

The scope of pathology is an ever-changing field, with continuous evolution in the demands to train a competent surgical pathologist. Surgical pathology rotations have successfully trained experts in the gross examination and dissection of surgical specimens and the diagnosis and workup of routine and complex cases. The Accreditation Council for Graduate Medical Education (ACGME) has set standards and expectations for pathology residency programs, leading to greater oversight, standardization, and scrutiny to train proficient pathologists to enter the workforce.^1^ Additionally, milestones were adopted to monitor resident progression throughout their training.^2^^,^^3^ While the ACGME has broadly advocated for these quality control measures, only one published study has reported quality improvement in a surgical pathology resident rotation curriculum by implementing a point system to limit grossing an excess volume of cases.^4^

The surgical pathology rotation forms the cornerstone of Anatomic Pathology training, comprising the majority of rotations a pathology trainee will experience. Throughout the United States, there is no set standard on how a surgical pathology rotation should be structured, and many different institutions follow different rotation schemes that fit the model and function of their Pathology department. Numerous factors are considered when designing surgical pathology rotations, including dependence on pathology residents as grossers, staffing and adequacy of pathology assistants, case volume, frozen section coverage, and general vs. subspecialty sign-out systems.

Traditionally, surgical pathology rotations are broken down by “cycles” of days. They tend to be institution-specific, taking into consideration the unique features of each pathology department with how residents integrate into the dependent natures of the pathology workflow. While each institution tends to have different variations of surgical pathology cycles, the preeminent current cycles are notably a “1-day cycle” and a “3-day cycle”. In the “1-day cycle”, residents spend the day previewing cases and following up on ancillary studies, sitting with attending pathologists for sign-out, covering frozen sections, and grossing specimens on a daily basis, tending to stay on a specific subspecialty for the entire week at a time. A “3-day cycle” usually consists of 1 day of grossing, 1 day of frozens, and 1 day of sign-out. Previewing cases may occur during the frozen or sign-out days, although the latter may be preferred at many institutions to minimize turnaround time. The frequency of changing between subspecialties is program-dependent, with many opting to switch specialties following each cycle of 3-days to gain maximum exposure to specimen types. While many models exist, there is no consensus on which best fits the goals and educational needs of a pathology trainee.

Contemporary pathology trainees at major academic centers face unique challenges that threaten the educational experience in surgical pathology. Nationwide workplace shortages, notably in histology and immunohistochemistry, have caused increased delays in case distribution compounded by increased specimen volumes, leading to heightened caseloads for histology processing.^5^ These issues were exacerbated by the COVID-19 pandemic, worsening the workforce shortage in laboratory medicine. These delays have reduced the preview time of cases for trainees, setting the stage for significant issues in trainee education and development.

In our institution, we observed that our traditional “3-day-cycle” consistently and negatively impacted resident education by reducing preview time in the setting of histology delays given the staffing issues brought on during the COVID pandemic, case follow-up, immunohistochemical (IHC) interpretation, and ancillary and molecular study interpretation. In order to meet the rising demands of a surgical pathologist, we aimed to innovate and modernize our surgical pathology rotation to improve resident self-performance assessment, satisfaction, education, and performance in internal quality control metrics. While few studies have aimed to improve the surgical pathology introductory experience through improved training boot camp months, to date, no such quality improvement studies have been undertaken for a complete overhaul of surgical pathology training.^6^^,^^7^

## Materials and methods

This study is Institutional Review Board exempt. A systematic and holistic review of the surgical pathology rotation was undertaken over a one-year period. Rotation evaluations submitted by trainees formed the cornerstone of this process, as well as utilization of the yearly ACGME program review, formalized feedback at Education Committee Meetings, six-month resident meetings, and informal feedback at Resident Meetings. The review process was performed by the director of the surgical pathology rotation in a large tertiary academic hospital, with assistance from senior pathologists with specializations in education and resident development, as well as pathology trainees interested in quality improvement. Aggregates of resident and faculty rotation evaluations were reviewed, and specific deficiencies in the 3-day cycle (grossing, frozens/preview, sign-out) were identified. In addition, qualitative data analysis by recurring resident and faculty comments and objective data through rotation evaluation scoring were utilized to highlight critical components consistently deemed deficient in the current rotation model. A review of nationwide pathology residency surgical pathology rotations was also performed, including extensive literature searches of quality improvement in Anatomic Pathology training, observation of other residency program rotation models, and investigation of resident dependence on Anatomic Pathology workflow (Table 1^4^, ^5^, ^6^, ^7^, ^8^, ^9^).

**Table 1:** Literature regarding quality improvement in anatomic pathology.

Author	Subject	Improvement
Mehr et al.^4^	Surgical Pathology	Point cap system Restructuring
Furlong et al.^6^	Surgical Pathology	Bootcamp rotation
Smith et al.^7^	Surgical Pathology	Bootcamp rotation-Military
Sinard et al.^8^	Autopsy	Increased visibility Service reporting Quality of data
Mai et al.^9^	Anatomic Pathology	Complete program overhaul Two preliminary years Two subspecialty years

The pathology residency class comprised 25 residents, of which 23 were AP/CP track, and 18 were included in the study as those had experience under the prior rotation structure. Characteristics of the surgical pathology rotation that were notable to our institution included resident dependence in gross room workflow, a four grossing bench system (Breast, Gynecologic and Perinatal, Gastrointestinal and Genitourinary, and General Surgical Pathology), and resident dependence for frozen sections.

An innovative surgical pathology rotation was proposed, composed of a 6-day cycle, consisting of 2 grossing days, 1 frozens/biopsies/preview day, 2 dedicated sign-out days for grossed cases, and 1 frozen/biopsies/case completion day (Table 2). Additionally, rotation tracks were created to ensure equal exposure to subspecialties throughout training, with residents assigned to one track per block (Table 3). The rotation was developed utilizing the core tenants of the previous 3-day cycle (i.e., grossing/frozens/sign-out), which were engrained into the program's Anatomic Pathology department workflow considering the resident dependence on grossing and frozen coverage. The director of surgical pathology created the overall rotation structure, with input from subspecialty division directors, laboratory leadership in Anatomic Pathology, pathology assistants, and gross room staff. Total grossing, sign-out, and frozen days remained identical to the previous 3-day cycle but were rearranged to maximize resident exposure and completion of cases while maintaining an identical workload for the laboratory staff, pathology assistants, and trainees. Grossing days and sign-out days were paired (2 consecutive days), with a frozen day separating them (ex. Grossing-grossing-frozen-sign out-sign out). The six-day cycle accommodates potential histology delays by allowing an additional “buffer” day for case processing, retrieval, preview, and self-studying on subspecialty topics. A second frozen day at the tail-end of the 6-day cycle was proposed to see all cases to completion by wrapping up IHC and ancillary studies. Graduated responsibility was increased by tasking residents to triage all cases and order ancillary studies, including IHC, special stains, and molecular studies independently, with appropriate guidance from the case attending pathologist as warranted. Proper coding and billing were emphasized, placing residents in charge of addressing and correcting coding issues. Surgical teams were notified of potentially increased turnaround times for cases; however, no concerns were brought to the attention of the pathology department.

**Table 2:** Comparison of the surgical pathology rotation in a typical 4-week block.

Day	Previous Rotation	New Rotation
1	Gross GSP	Gross GSP
2	Frozens/Preview	Gross GSP/Preview
3	Sign-out GSP	Frozens/Preview/Bx
4	Gross GI/GU	Sign-out GSP/Follow-up IHC
5	Frozens/Preview	Sign-out GSP/Follow-up IHC
6	Sign-out GI/GU	Frozens/Follow-up IHC/Bx
7	Gross GYN/Placenta	Gross GI/GU
8	Frozens/Preview	Gross GI/GU/Preview
9	Sign-out GYN/Placenta	Frozens/Preview/Bx
10	Gross Breast	Sign-out GI/GU/Follow-up IHC
11	Frozens/Preview	Sign-out GI/GU/Follow-up IHC
12	Sign-out Breast	Frozens/Follow-up IHC/Bx
13	Gross GSP	Gross GYN-Placenta
14	Frozens/Preview	Gross GYN/Preview
15	Sign-out GSP	Frozens/Preview/Bx
16	Gross GI/GU	Sign-out GYN-Placenta/Follow-up IHC
17	Frozens/Preview	Sign-out GYN-Placenta/Follow-up IHC
18	Sign-out GI/GU	Frozens/Follow-up IHC/Bx
19	Gross GYN-Placenta	Gross Breast
20	Frozens/Preview	Gross Breast

Abbreviations: GSP-General Surgical Pathology. GI-Gastrointestinal. GU-Genitourinary. GYN-Gynecologic. IHC-Immunohistochemistry. Bx-Biopsies.

**Table 3:** Track listings for each individual resident in a typing block rotation.

Day	T1	T2	T3	T4	T5	T6
1	Gross Gyn-Pla	Frozens/Preview Breast	Sign-out GI-GU/Follow-up IHC	Gross GI-GU	Frozens/Preview/Bx	Sign-out Gyn-Pla/Follow-up IHC
2	Gross Gyn-Pla/Preview	Sign-out Breast/Follow-up IHC	Frozens/Follow-up IHC/Bx	Gross GI-GU/Preview	Sign-out GSP/Follow-up IHC	Frozens/Follow-up IHC/Bx
3	Frozens/Preview/Bx	Sign-out Breast/Follow-up IHC	Gross GSP	Frozens/Preview/Bx	Sign-out GSP/Follow-up IHC	Gross Breast
4	Sign-out Gyn-Pla/Follow-up IHC	Frozens/Follow-up IHC/Bx	Gross GSP/Preview	Sign-out GI-GU/Follow-up IHC	Frozens/Follow-up IHC/Bx	Gross Breast/Preview
5	Sign-out Gyn-Pla/Follow-up IHC	Gross Gyn-Pla	Frozens/Preview/Bx	Sign-out GI-GU/Follow-up IHC	Gross GI-GU	Frozens/Preview/Bx
6	Frozens/Follow-up IHC/Bx	Gross Gyn-Pla/Preview	Sign-out GSP/Follow-up IHC	Frozens/Follow-up IHC/Bx	Gross GI-GU/Preview	Sign-out Breast/Follow-up IHC
7	Gross GSP	Frozens/Preview/Bx	Sign-out GSP/Follow-up IHC	Gross Breast	Frozens/Preview/Bx	Sign-out Breast/Follow-up IHC
8	Gross GSP/Preview	Sign-out Gyn-Pla/Follow-up IHC	Frozens/Follow-up IHC/Bx	Gross Breast/Preview	Sign-out GI-GU/Follow-up IHC	Frozens/Follow-up IHC/Bx
9	Frozens/Preview/Bx	Sign-out Gyn-Pla/Follow-up IHC	Gross Gyn-Pla	Frozens/Preview/Bx	Sign-out GI-GU/Follow-up IHC	Gross GI-GU
10	Sign-out GSP/Follow-up IHC	Frozens/Follow-up IHC/Bx	Gross Gyn-Pla/Preview	Sign-out Breast/Follow-up IHC	Frozens/Follow-up IHC/Bx	Gross GI-GU/Preview
11	Sign-out GSP/Follow-up IHC	Gross GSP	Frozens/Preview/Bx	Sign-out Breast/Follow-up IHC	Gross Breast	Frozens/Preview/Bx
12	Frozens/Follow-up IHC/Bx	Gross GSP/Preview	Sign-out Gyn-Pla/Follow-up IHC	Frozens/Follow-up IHC/Bx	Gross Breast/Preview	Sign-out GI-GU/Follow-up IHC
13	Gross GI-GU	Frozens/Preview/Bx	Sign-out Gyn-Pla/Follow-up IHC	Gross Gyn-Pla	Frozens/Preview/Bx	Sign-out GI-GU/Follow-up IHC
14	Gross GI-GU/Preview	Sign-out GSP/Follow-up IHC	Frozens/Follow-up IHC/Bx	Gross Gyn-Pla/Preview	Sign-out Breast/Follow-up IHC	Frozens/Follow-up IHC/Bx
15	Frozens/Preview/Bx	Sign-out GSP/Follow-up IHC	Gross Breast	Frozens/Preview/Bx	Sign-out Breast/Follow-up IHC	Gross GSP
16	Sign-out GI-GU/Follow-up IHC	Frozens/Follow-up IHC/Bx	Gross Breast/Preview	Sign-out Gyn-Pla/Follow-up IHC	Frozens/Follow-up IHC/Bx	Gross GSP/Preview
17	Sign-out GI-GU/Follow-up IHC	Gross GI-GU/Preview	Frozens/Preview/Bx	Sign-out Gyn-Pla/Follow-up IHC	Gross Gyn-Pla	Frozens/Preview/Bx
18	Frozens/Follow-up IHC/Bx	Gross GI-GU/Preview	Sign-out Breast/Follow-up IHC	Frozens/Follow-up IHC/Bx	Gross Gyn-Pla/Preview	Sign-out GSP/Follow-up IHC
19	Gross Breast	Frozens/Preview/Bx	Sign-out Breast/Follow-up IHC	Gross GSP	Frozens/Preview/Bx	Sign-out GSP/Follow-up IHC
20	Gross Breast	Sign-out GI-GU/Follow-up IHC	Frozens/Follow-up IHC/Bx	Gross GSP	Sign-out Gyn-Pla/Follow-up IHC	Frozens/Follow-up IHC/Bx

Abbreviations: GSP-General Surgical Pathology. GI-Gastrointestinal. GU-Genitourinary. GYN-Gynecologic, Pla-Placenta. IHC-Immunohistochemistry. Bx-Biopsies. T-Track.

To track the residents' perception of training and satisfaction with the new rotation cycle, a Google Forms survey was created and distributed to the 18 pathology residents included in the initial cohort prior to implementation and six months after the new rotation model implementation. The survey consisted of the ACGME milestones for levels 4 and 5 related to surgical pathology (Patient Care 1: Reporting (PC1), Patient Care 2: Grossing (PC2), Patient Care 3: Clinical Consultation (PC3), Patient Care 4: Interpretation and Diagnosis (PC4), Patient Care 5: Intra-operative Consultation (PC5), Medical Knowledge 1: Diagnostic Knowledge (MK1), Medical Knowledge 2: Clinical Reasoning (MK2)) (Table 4). Only level 4 and level 5 milestones were utilized, as level 4 is a graduation target for a pathology trainee and is what the pathology trainee should aspire to reach before fellowship or independent practice. Therefore, while the other milestone levels are necessary to track development, we aimed to have our endpoint at graduation level. In addition, assessments via the same resident self-reported Google Form regarding internal rotation quality metrics of interest were included: adequacy of specimen fixation, time spent grossing, when residents were receiving cases, adequacy of preview time, reviewing IHC, graduated responsibility, and preparedness for fellowship (Table 5). The residents that experienced the rotation transition completed the survey before implementing the new rotation model and subsequently completed the survey after experiencing the new rotation model, 6 months later. Scores on agreeability of the metrics were evaluated on a 5-point Likert scale, where score 1 was “Strongly Disagree” and score 5 was “Strongly Agree. ”

**Table 4:** List of assessed ACGME milestones.

ACGME Milestones	Resident Performance
PC1-Level 4	- Independently generates timely integrated reports for complex cases. - Generates an amended/addended report and documents communication with the clinical team, as appropriate - Independently generates a report that includes the language of uncertainty and complex recommendations
PC1-Level 5	- Independently generates a nuanced report that expresses the ambiguity and uncertainty for a complex case
PC2-Level 4	- Independently triages, samples, and documents complex cases - Independently resolves specimen integrity issues, as needed - Efficiently finishes own workload and assists others as needed
PC2-Level 5	- Applies innovative approaches of grossing to demonstrate optimal pathology in unique specimens - Serves as an expert for gross examination
PC3-Level 4	- Manages complex consultations independently
PC3-Level 5	- Recognized as an expert in providing comprehensive consultations
PC4-Level 4	- Makes accurate diagnoses and interpretations of test results - Gives consideration to confounding factors in formulating an interpretation(s) and diagnoses - Recommends further work-up using diagnostic algorithms and recommends therapeutic options, as appropriate
PC4-Level 5	- Is an expert diagnostician - Proposes optimal diagnostic and therapeutic strategies based on patterns within a population
PC5-Level 4	- For complex cases, independently manages and addresses requests for IOC - Supervises junior residents and advises technical staff members in the performance of IOC - Independently interprets and communicates IOC/FS and correlates with final diagnosis in routine cases and in some complex cases
PC5-Level 5	- Expertly manages all IOC
MK1-Level 4	- Integrates advanced knowledge of anatomic, cellular, and molecular pathology to common and uncommon diagnoses
MK1-Level 5	- Recognized as an expert in the integration of anatomic, cellular, and molecular pathology knowledge to disease
MK2-Level 4	- Independently synthesizes information to inform clinical reasoning in complex cases - Independently seeks out, analyzes, and applies relevant original research to diagnostic decision making in complex clinical cases
MK1-Level 5	- Demonstrates intuitive approach to clinical reasoning for complex cases

Abbreviations: PC-Patient Care. MK-Medical Knowledge. Adapted from: Accreditation Council for Graduate Medical Education. ACGME Pathology Milestones. ACGME. Published February 2019. https://www.acgme.org/globalassets/pdfs/milestones/pathologymilestones.pdf.^3^

**Table 5:** Internal quality control metrics assessed.

Internal Quality Metrics
I can adequately fix all specimens during my normal grossing day.
I routinely gross over the PGY specific point cap
I routinely gross past 6 p.m. on my grossing days.
I receive my cases the day prior to sign-out.
I receive my cases the day of sign-out.
I have adequate time to preview my cases.
I routinely review immunohistochemical stains ordered on specimens I gross.
There is enough graduated responsibility on the surgical pathology rotation.
The surgical pathology rotation will prepare me well for independent practice/fellowship

Additionally, to obtain objective data on the new rotation, Clinical Competency Committee (CCC) evaluations of the milestones mentioned earlier for each resident were assessed in paired PGY levels for both the 3-day cycle and 6-day cycle. The CCC evaluations available for review included 19 residents for the 3-day cycle (PGY1: 6, PGY2: 4, PGY3: 5, and PGY4: 4) and 22 residents for the 6-day cycle (PGY1: 5, PGY2: 7, PGY3: 5, and PGY4: 5).

Pre- and post-implementation survey responses were tracked and compared utilizing GraphPad Prism v9 (GraphPad Software, San Diego, CA). Statistical significance was calculated via multiple unpaired t-tests. P-value thresholds were calculated as follows: ∗*P* < .05, ∗∗*P* < .01, ∗∗∗*P* < .001.

## Results

Internal quality issues with the original surgical pathology rotation were identified during the review of rotation evaluations. Histology delays, which many departments face, were noted to limit case preview time severely. There was a lack of proper workup and interpretation of complex cases by limited IHC and ancillary study review as residents proceeded to busy grossing days immediately after sign-out. Additionally, there was limited graduated responsibility as residents at all postgraduate levels were not independently triaging cases and ordering IHC.

The pre-intervention survey was completed by residents at postgraduate levels PGY1, 2, and 3 at the end of the academic year (n = 18). In the old surgical pathology rotation structure, most residents across each PGY year disagreed or strongly disagreed (below tier 3 agreement) that they reached ACGME levels 4 and 5 for milestones PC1, PC2, PC4, PC5, MK1, and MK2. The internal quality metrics that residents viewed as insufficient included: adequate time to fix specimens, receiving slides early for adequate preview, time to review IHC, graduated responsibility, and readiness for independent practice. The post-implementation survey was completed by the same cohort of residents, now corresponding to postgraduate levels PGY2, 3, and 4 (n = 15, of which 3 were lost in follow-up).

**Fig. 1:**
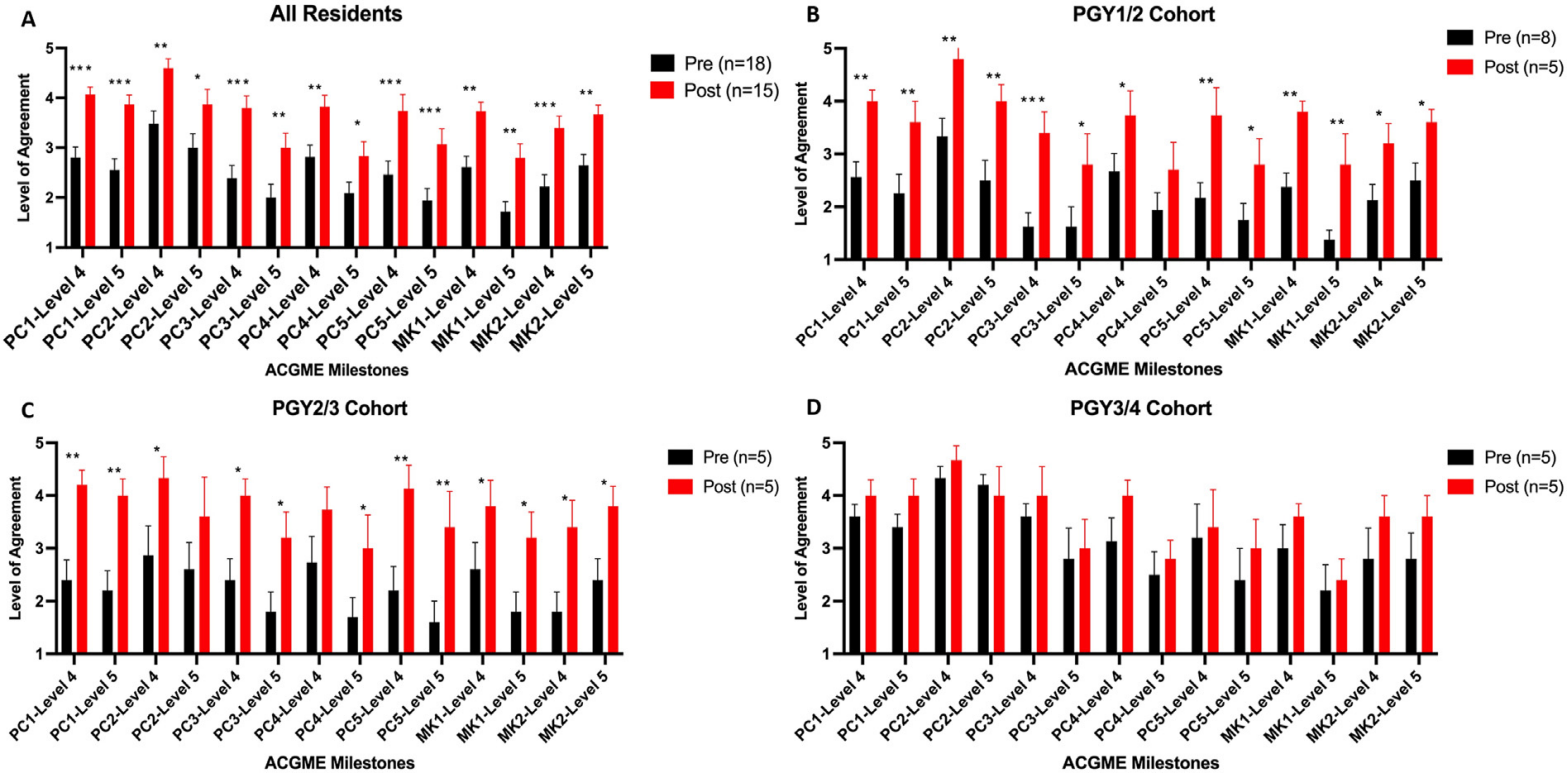
ACGME milestones level 4 and 5 significantly improve post-6-day cycle implementation. All residents (A), PGY1-to-PGY2 cohort (B), PGY2-to-PGY3 cohort (C), and PGY3-to-PGY4 cohort (D). Abbreviations: PC-Patient Care. MK-Medical Knowledge. ∗*P* < .05, ∗∗*P* < .01, ∗∗∗*P* < .001.

After the implementation of the revised curriculum, when the entire cohort of residents was compared together, there was a statistically significant increase in agreement across all ACGME level 4 and 5 milestones (Fig. 1A, Supplemental Table 1). The PGY1-to-PGY2 cohort significantly increased agreement across all ACGME level 4 and 5 milestones except for PC4-level 5 (Fig. 1B, Supplemental Table 2). In addition, the PGY2-to-PGY3 cohort significantly increased agreement across all ACGME level 4 and 5 milestones, except for PC2-level 5 and PC4-level 4 (Fig. 1C, Supplemental Table 3). After implementing the revised surgical pathology curriculum, the PGY3-to-PGY4 cohort had no significant changes in ACGME milestones (Fig. 1D, Supplemental Table 4).

**Fig. 2:**
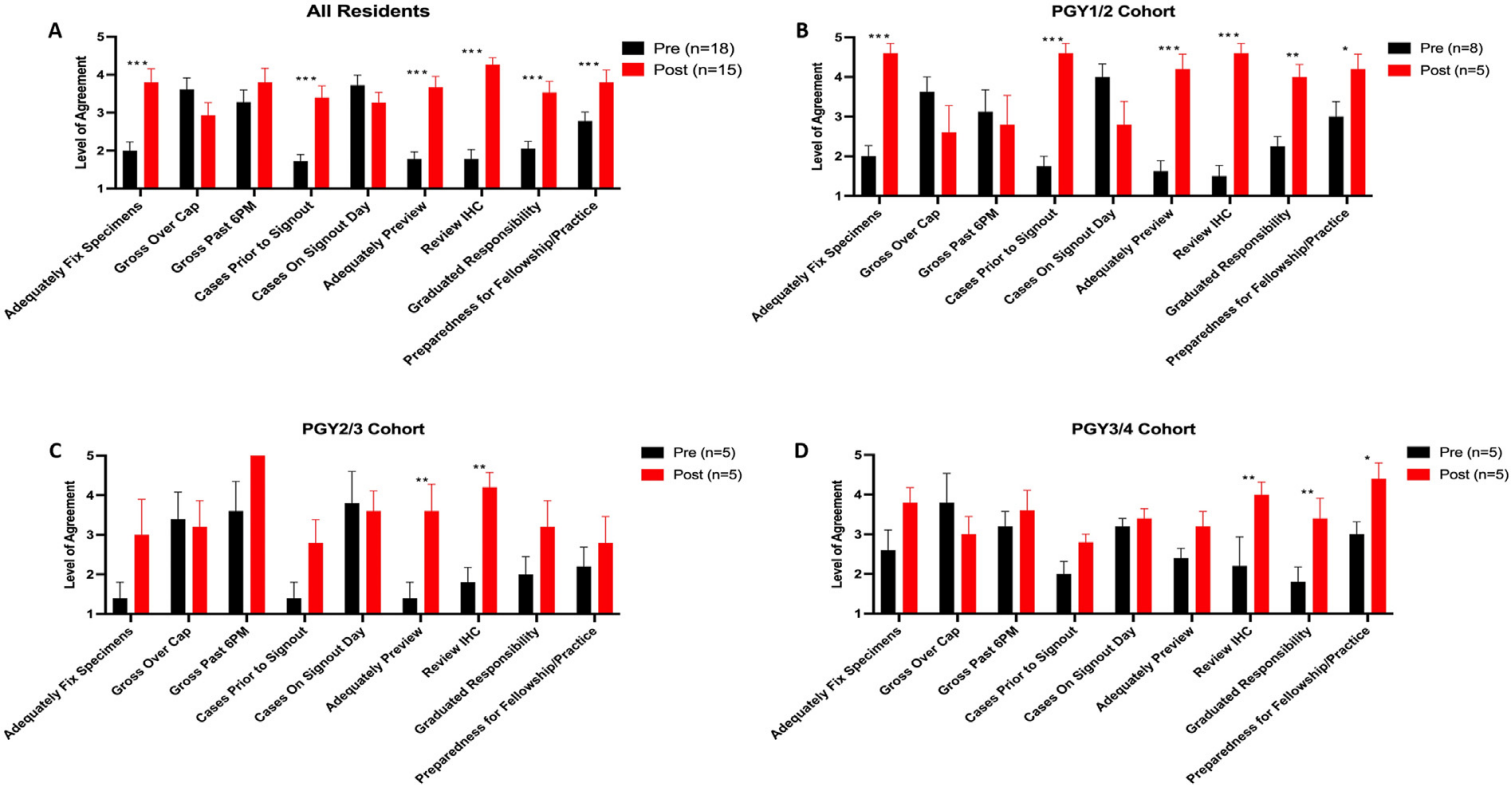
Internal quality metrics significantly increase post 6-day cycle implementation. All residents (A), PGY1-to-PGY2 cohort (B), PGY2-to-PGY3 cohort (C), and PGY3-to-PGY4 cohort (D). ∗*P* < .05, ∗∗*P* < .01, ∗∗∗*P* < .001.

Internal quality control metrics that significantly increased across the entire cohort were the ability to adequately fix specimens, receiving cases prior to sign-out, adequate time to preview cases, reviewing IHC, appropriate graduated responsibility, and preparedness for fellowship (*P* < .001) (Fig. 2A, Supplemental Table 5). The PGY1-to-PGY2 cohort had significant increases in the same domains as the entire residency cohort (Fig. 2B, Supplemental Table 6). The PGY2-to-PGY3 cohort noted only significant improvements in the adequacy of preview time and reviewing of IHC (*P* < .01) (Fig. 2C, Supplemental Table 7). The PGY3-to-PGY4 cohort demonstrated improvement in reviewing IHC, the level of graduated responsibility, and preparedness for fellowship (*P* < .05 and *P* < .01, respectively) (Fig. 2D, Supplemental Table 8).

**Fig. 3:**
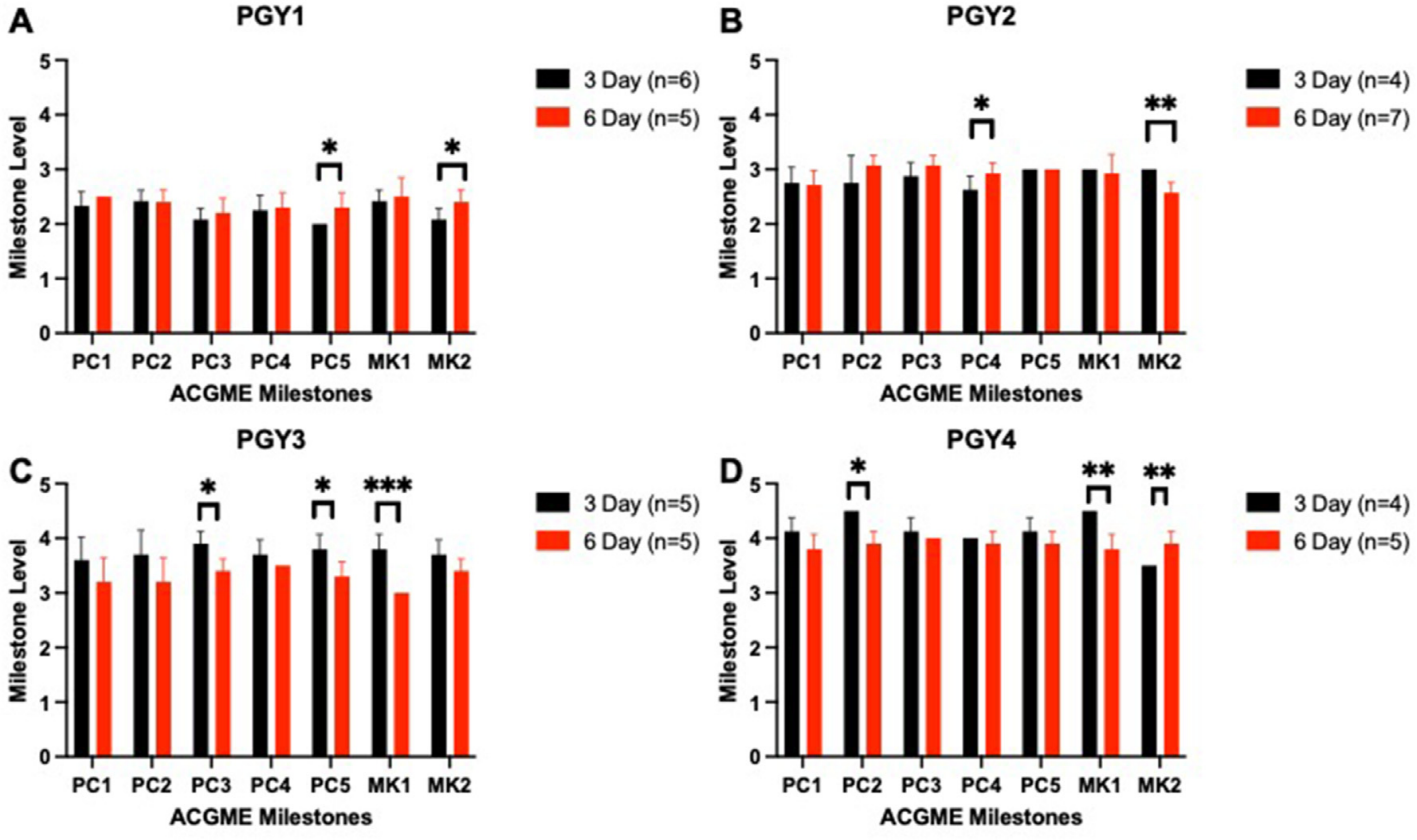
Clinical Competency Committee performance via paired PGY cohorts. PGY1 (A), PGY2(B), PGY3(C), and PGY4(D). ∗*P* < .05, ∗∗*P* < .01, ∗∗∗*P* < .001.

CCC assessments by PGY levels revealed an overall trend towards improvement in the PGY1 class, with statistically significant increases in both PC5 and MK2 (Fig. 3A, Supplemental Table 9). The PGY2 classes had trends towards improvement within the 6-day cycle, with significant increases in PC4 but decreased in MK2 (Fig. 3B, Supplemental Table 10). The PGY3 classes maintained overall levels but decreased in PC3, PC5, and MK1 (Fig. 3C, Supplemental Table 11). Finally, the PGY4 classes had overall maintained levels with decreases in PC2 and MK1 but increase in MK2 (Fig. 3D, Supplemental Table 12). Overall, 78.6% (22/28) of total milestones across the different PGY levels showed either maintained performance, trending towards increased performance, or significantly increased performance when comparing the 6-day cycle to the 3-day cycle.

No duty hour violations were reported in either the 3-day cycle or the 6-day cycle. Residents ' pre-implementation turnaround time (TAT) and IHC ordering was not tracked in the Cerner® laboratory information services. The post-implementation, coinciding with the switch to Epic Beaker®, TAT was as follows: 4.97 ± 0.22 days (PGY1-2 cohort), 4.96 ± 0.18 days (PGY2-3 cohort), 5.18 ± 0.17 days (PGY3-4 cohort), and 5.03 ± 0.11 days (all resident). Overall IHC and special stains ordered by residents post-implementation per surgical pathology block rotation was: 89.11 ± 10.73 (PGY1-2 cohort), 118.67 ± 6.87 (PGY2-3 cohort), 142.5 ± 34.03 (PGY3-4 cohort), and 109.65 ± 9.59 (all residents).

## Discussion

Herein, we present a novel surgical pathology rotation cycle that meets the demands of a large academic pathology department. Consistent quality improvement to better the education demands of residents is of utmost importance in the expanding scope of pathology. While the ACGME sets guidelines for surgical pathology training, consensus and nationwide reproducibility of rotation structures are still lacking.^8^ Although quality improvement projects in restructuring other core pathology rotations, such as autopsy, or general program overhauls, have been previously described, surgical pathology consistently lacks quality improvement measures.^4^^,^^8^^,^^9^

Most notably, in our previous 3-day cycle of the surgical pathology rotation, numerous external and internal factors severely impacted resident education. The shortage of histotechnologists limited case processing and distribution, as evident in the disagreement of residents on previewing time. Additionally, proceeding directly to a grossing day after a sign-out day limited IHC review, case follow-up, and graduated responsibility. By assessing these internal quality metrics through self-assessment surveys, the implementation of the 6-day cycle rectified these issues within our program, as evident in the statistically significant increase in the assessed metrics. Residents expressed satisfaction and agreement in multiple levels of quality metrics, including having ample time for previewing by receiving cases on time, reviewing IHC, and self-assessed preparedness for fellowship with graduated responsibility.

By showing these quality improvements, the 6-day cycle is a cornerstone for our anatomic pathology rotation.

Resident self-assessment performance, as indicated by increased agreement in the subset of ACGME milestones assessed, showed improvement over the implementation of the 6-day surgical pathology cycle. When assessed as a general cohort of total residents, our residents conveyed an increased agreement in reaching ACGME milestones relating to surgical pathology. The PGY1-to-2 cohort demonstrated significant improvements in all assessed milestones except for PC4-level 5. Likewise, the PGY2-to-3 cohort expressed significant improvements in all milestones except for PC2-level 5 and PC4-level 4. While the PGY3-to-4 cohort conveyed increases in all milestones, none were statistically significant. With burnout occurring at an increased rate in pathology trainees directly related to job stress and feelings of inadequacy, our revised 6-day cycle exemplifies a potential method of quality improvement to tackle these issues head-on.^10^, ^11^, ^12^ While burnout was not directly addressed, the surveyed improvements suggest that the 6-day cycle implementation can improve the adequacy and satisfaction of our surgical pathology education.

Clinical Competency Committee (CCC) evaluations were also assessed through paired PGY levels from the prior 3-day- to 6-day cycles. Overall, these trended toward similar, mildly increased, and significantly increased levels in most of the milestones assessed. Notably, the CCC data showed the most improvement in the PGY1 paired classes, highlighting an educational benefit for the most junior residents to guide them in their surgical pathology training. When analyzing the CCC data, the 6-day cycle shows stable progression to the 3-day cycle. Coupled with the high resident satisfaction concerning internal quality metrics, the 6-day cycle improved the overall surgical pathology rotation experience.

The modern pathology resident faces increased challenges when entering the workforce as an attending physician, which we aimed to address through our new rotation cycle. In addition, recent graduates have indicated they aim to enhance pathology skills not taught during residency, with a perceived lack of readiness for independent practice.^13^ We aimed to address this by providing heightened autonomy in our rotation cycle to simulate as close to independent practice as possible within the constraints of the departmental organization. Our cohort within the 6-day cycle demonstrated significant increases in agreement on readiness for independent practice, highlighting the satisfaction of the current model.

Additionally, recent reports on qualities that pathology employers seek and those in which pathology trainees believe they are deficient include areas of billing/coding, laboratory management, and molecular pathology, all of which are aspects of graduated responsibility in pathology training.^14^^,^^15^ While not directly assessed in our survey, in the 6-day cycle, residents are taught the aspects of billing and are expected to address and correct coding issues with cases. Additionally, residents are the point person for all laboratory issues, and residents triage for molecular studies, including fluorescent in-situ hybridization (FISH), next-generation sequencing, and methylation profiling. Furthermore, deficiencies in IHC interpretation in new graduates were also noted.^16^ However, as evident in the internal quality control metrics, there was a statistically significant improvement in abilities to view IHC, presumably leading to improved abilities of IHC interpretation for the accurate competition of cases.

The revised 6-day cycle allows our residents to have the opportunity to take ownership of their cases, troubleshooting all laboratory issues, from the gross room to histology to IHC. The resident performs proper IHC ordering and interpretation. While not tracked secondary to LIS limitations before the implementation of the 6-day cycle, residents averaged ordering ∼109 stains per rotation after the implementation of the 6-day cycle, likely an increase based on the observational comparison.

Although the revised surgical pathology rotation has significantly improved resident education and milestone performance, potential issues remain in surgical pathology training. Given the increased time length to finalize cases, turnaround time may be impacted slightly; however, the surgical teams were previously notified of the 6-day cycle prior to its implementation. While TAT was not tracked prior to the implementation of the 6-day cycle, the average TAT for resident grossed cases was 5.02 days, which considering the additional buffer day added into the 6-day cycle from the prior 3-day cycle, likely indicates ∼1 day additional TAT for resident grossed cases. Additionally, during a 4-week rotation, each resident participates in ∼3 benches, rather than experiencing all four benches multiple times, necessitating residents to keep track of which benches they missed for future rotation planning. Notably, residents are solely responsible for their grossed cases, meaning Pathology Assistants-grossed cases and potential additional learning material goes unseen without additional motivation by the faculty or the trainees.

Future changes and new directions to our novel 6-day cycle of surgical pathology are ongoing. Notably, the implementation of a senior “pre-fellow” rotation in which the senior resident acts as a fellow: signing out the entire volume of PA grossed cases and outside consults without grossing in their last two rotations of surgical pathology training. Additionally, entrustable professional activities (EPAs) are currently being implemented as a new methodology to track each resident's development individually.

The 6-day surgical pathology rotation implementation in our institution improved the trainee's self-assessments of competency, satisfaction, and exposure to the specialty without requiring additional undue burden on laboratory staff, their assigned workload, or resources. To the best of our knowledge, this innovative design is unique to our institution and meets the unique demands and characteristics of our Anatomic Pathology department. After pre- and post-implementation surveys, there were statistically significant improvements in both self-assessed performance and internal quality metrics relating to satisfaction and strength of training, leading to the enhancement of resident education. Additionally, the CCC data of paired PGY levels showed similar levels of progression both pre-and post-implementation, with increases mainly seen in the PGY1 class, highlighting a potential benefit to incoming residents. Notably, this study does have a few limitations. The reported self-improvement might have been due to a natural gain of confidence over six months of training rather than an actual improvement solely attributed to the 6-day-cycle implementation. In analyzing the CCC data, there is a low overall number of total residents assessed, given the short time the 6-day cycle has been in place. Future studies incorporating the data of new residents will be done to continue to analyze the progression and quality of the rotation.

## Conclusions

Our new surgical pathology rotation can be a framework for other residency programs, aiming to train competent and independent pathology consultants ready to enter practice. The novel 6-day surgical pathology rotational model is a modern approach to training in academic centers with improvement in resident satisfaction, quality control metrics, and does not place undue burden on trainees, staff, and the department.

## Funding

The article processing fee for this article was funded by an Open Access Award given by the Society of ‘67, which supports the mission of the Association of Pathology Chairs to produce the next generation of outstanding investigators and educational scholars in the field of pathology. This award helps to promote the publication of high-quality original scholarship in Academic Pathology by authors at an early stage of academic development.

## Declaration of interests

The authors declare that they have no known competing financial interests or personal relationships that could have appeared to influence the work reported in this paper.
