# Supplementary material for: A novel 6-day cycle surgical pathology rotation improves resident satisfaction and maintains Accreditation Council for Graduate Medical Education (ACGME) milestone performance
Source: Acad Pathol. 2023 Jun 30;10(3):100088. doi: 10.1016/j.acpath.2023.100088 (PMC10336254; doi:10.1016/j.acpath.2023.100088)
Supplement: Multimedia component 1 [file mmc1.docx]

Supplemental Table 1: ACGME milestone agreements across all residents

| Milestone | Mean Agreement | *P** |
| --- | --- | --- |
| PC1-Level 4 | 2.806  4.067 | <.001 |
| PC1-Level 5 | 2.556  3.867 | <.001 |
| PC2-Level 4 | 3.481  4.600 | .0014 |
| PC2-Level 5 | 3.000  3.867 | .013 |
| PC3-Level 4 | 2.389  3.800 | <.001 |
| PC3-Level 5 | 2.000  3.000 | .0042 |
| PC4-Level 4 | 2.815  3.822 | .0040 |
| PC4-Level 5 | 2.088  2.833 | .033 |
| PC5-Level 4 | 2.463  3.738 | <.001 |
| PC5-Level 5 | 1.941  3.067 | <.001 |
| MK1-Level 4 | 2.611  3.733 | .0013 |
| MK1-Level 5 | 1.722  2.800 | .0021 |
| MK2-Level 4 | 2.222  3.400 | <.001 |
| MK2-Level 5 | 2.647  3.667 | .0036 |

^*^Comparison of agreement from pre- and post- implementation surveys
