# Supplementary material for: A novel 6-day cycle surgical pathology rotation improves resident satisfaction and maintains Accreditation Council for Graduate Medical Education (ACGME) milestone performance
Source: Acad Pathol. 2023 Jun 30;10(3):100088. doi: 10.1016/j.acpath.2023.100088 (PMC10336254; doi:10.1016/j.acpath.2023.100088)
Supplement: Multimedia component 2 [file mmc2.docx]

Supplemental Table 2: ACGME milestone agreements across PGY1-PGY2 cohort

| Milestone | Mean Agreement | *P** |
| --- | --- | --- |
| PC1-Level 4 | 2.563  4.000 | .0061 |
| PC1-Level 5 | 2.250  3.600 | .0099 |
| PC2-Level 4 | 3.333  4.800 | .0051 |
| PC2-Level 5 | 2.500  4.000 | .0042 |
| PC3-Level 4 | 1.625  3.400 | <.001 |
| PC3-Level 5 | 1.625  2.800 | .024 |
| PC4-Level 4 | 2.667  3.733 | .041 |
| PC4-Level 5 | 1.938  2.700 | .14 |
| PC5-Level 4 | 2.167  3.733 | .0029 |
| PC5-Level 5 | 1.750  2.800 | .044 |
| MK1-Level 4 | 2.375  3.800 | .0065 |
| MK1-Level 5 | 1.375  2.800 | .0065 |
| MK2-Level 4 | 2.125  3.200 | .039 |
| MK2-Level 5 | 2.500  3.600 | .035 |

^*^Comparison of agreement from pre- and post- implementation surveys
