# Supplementary material for: A novel 6-day cycle surgical pathology rotation improves resident satisfaction and maintains Accreditation Council for Graduate Medical Education (ACGME) milestone performance
Source: Acad Pathol. 2023 Jun 30;10(3):100088. doi: 10.1016/j.acpath.2023.100088 (PMC10336254; doi:10.1016/j.acpath.2023.100088)
Supplement: Multimedia component 3 [file mmc3.docx]

Supplemental Table 3: ACGME milestone agreements across PGY2-PGY3 cohort

| Milestone | Mean Agreement | *P** |
| --- | --- | --- |
| PC1-Level 4 | 2.400  4.200 | .0068 |
| PC1-Level 5 | 2.200  4.000 | .0068 |
| PC2-Level 4 | 2.867  4.333 | .027 |
| PC2-Level 5 | 2.600  3.600 | .13 |
| PC3-Level 4 | 2.400  4.000 | .016 |
| PC3-Level 5 | 1.800  3.200 | .034 |
| PC4-Level 4 | 2.733  3.733 | .13 |
| PC4-Level 5 | 1.700  3.000 | .049 |
| PC5-Level 4 | 2.200  4.133 | .0037 |
| PC5-Level 5 | 1.600  3.400 | .0068 |
| MK1-Level 4 | 2.600  3.800 | .069 |
| MK1-Level 5 | 1.800  3.200 | .034 |
| MK2-Level 4 | 1.800  3.400 | .016 |
| MK2-Level 5 | 2.400  3.800 | .034 |

^*^Comparison of agreement from pre- and post- implementation surveys
