# Supplementary material for: A novel 6-day cycle surgical pathology rotation improves resident satisfaction and maintains Accreditation Council for Graduate Medical Education (ACGME) milestone performance
Source: Acad Pathol. 2023 Jun 30;10(3):100088. doi: 10.1016/j.acpath.2023.100088 (PMC10336254; doi:10.1016/j.acpath.2023.100088)
Supplement: Multimedia component 4 [file mmc4.docx]

Supplemental Table 4: ACGME milestone agreements across PGY3-PGY4 cohort

| Milestone | Mean Agreement | *P** |
| --- | --- | --- |
| PC1-Level 4 | 3.600  4.000 | .52 |
| PC1-Level 5 | 3.400  4.000 | .34 |
| PC2-Level 4 | 4.333  4.667 | .60 |
| PC2-Level 5 | 4.200  4.000 | .75 |
| PC3-Level 4 | 3.600  4.000 | .52 |
| PC3-Level 5 | 2.800  3.000 | .75 |
| PC4-Level 4 | 3.133  4.000 | .17 |
| PC4-Level 5 | 2.500  2.800 | .63 |
| PC5-Level 4 | 3.200  3.400 | .75 |
| PC5-Level 5 | 2.400  3.000 | .34 |
| MK1-Level 4 | 3.000  3.600 | .34 |
| MK1-Level 5 | 2.200  2.400 | .75 |
| MK2-Level 4 | 2.800  3.600 | .20 |
| MK2-Level 5 | 2.800  3.600 | .20 |

^*^Comparison of agreement from pre- and post- implementation surveys
