# Supplementary material for: A novel 6-day cycle surgical pathology rotation improves resident satisfaction and maintains Accreditation Council for Graduate Medical Education (ACGME) milestone performance
Source: Acad Pathol. 2023 Jun 30;10(3):100088. doi: 10.1016/j.acpath.2023.100088 (PMC10336254; doi:10.1016/j.acpath.2023.100088)
Supplement: Multimedia component 5 [file mmc5.docx]

Supplemental Table 5: Internal quality metric agreements across all residents

| Internal Metric | Mean Agreement | *P** |
| --- | --- | --- |
| Adequately Fix Specimens | 2.000  3.800 | <.001 |
| Gross Over Cap | 3.611  2.933 | .081 |
| Gross Past 6PM | 3.278  3.800 | .18 |
| Cases Prior to Signout | 1.722  3.400 | <.001 |
| Cases on Signout Day | 3.722  3.267 | .24 |
| Adequately Preview | 1.778  3.667 | <.001 |
| Review IHC | 1.778  4.267 | <.001 |
| Graduated Responsibility | 2.056  3.544 | <.001 |
| Preparedness for Practice | 2.778  3.800 | .0087 |

^*^Comparison of agreement from pre- and post- implementation surveys
