# Supplementary material for: A novel 6-day cycle surgical pathology rotation improves resident satisfaction and maintains Accreditation Council for Graduate Medical Education (ACGME) milestone performance
Source: Acad Pathol. 2023 Jun 30;10(3):100088. doi: 10.1016/j.acpath.2023.100088 (PMC10336254; doi:10.1016/j.acpath.2023.100088)
Supplement: Multimedia component 6 [file mmc6.docx]

Supplemental Table 6: Internal quality metric agreements across PGY1-PGY2 cohort

| Internal Metric | Mean Agreement | *P** |
| --- | --- | --- |
| Adequately Fix Specimens | 2.000  4.600 | <.001 |
| Gross Over Cap | 3.625  2.600 | .073 |
| Gross Past 6PM | 3.125  2.800 | .56 |
| Cases Prior to Signout | 1.750  4.600 | <.001 |
| Cases on Signout Day | 4.000  2.800 | .034 |
| Adequately Preview | 1.625  4.200 | <.001 |
| Review IHC | 1.500  4.600 | <.001 |
| Graduated Responsibility | 2.250  4.000 | .0023 |
| Preparedness for Practice | 3.000  4.200 | .035 |

^*^Comparison of agreement from pre- and post- implementation surveys
