# Supplementary material for: A novel 6-day cycle surgical pathology rotation improves resident satisfaction and maintains Accreditation Council for Graduate Medical Education (ACGME) milestone performance
Source: Acad Pathol. 2023 Jun 30;10(3):100088. doi: 10.1016/j.acpath.2023.100088 (PMC10336254; doi:10.1016/j.acpath.2023.100088)
Supplement: Multimedia component 7 [file mmc7.docx]

Supplemental Table 7: Internal quality metric agreements across PGY2-PGY3 cohort

| Internal Metric | Mean Agreement | *P** |
| --- | --- | --- |
| Adequately Fix Specimens | 1.400  3.000 | .055 |
| Gross Over Cap | 3.400  3.200 | .81 |
| Gross Past 6PM | 3.600  5.000 | .092 |
| Cases Prior to Signout | 1.400  2.800 | .092 |
| Cases on Signout Day | 3.800  3.600 | .81 |
| Adequately Preview | 1.400  3.600 | .0090 |
| Review IHC | 1.800  4.200 | .0045 |
| Graduated Responsibility | 2.000  3.200 | .15 |
| Preparedness for Practice | 2.200  2.800 | .47 |

^*^Comparison of agreement from pre- and post- implementation surveys
