# Supplementary material for: A novel 6-day cycle surgical pathology rotation improves resident satisfaction and maintains Accreditation Council for Graduate Medical Education (ACGME) milestone performance
Source: Acad Pathol. 2023 Jun 30;10(3):100088. doi: 10.1016/j.acpath.2023.100088 (PMC10336254; doi:10.1016/j.acpath.2023.100088)
Supplement: Multimedia component 8 [file mmc8.docx]

Supplemental Table 8: Internal quality metric agreements across PGY3-PGY4 cohort

| Internal Metric | Mean Agreement | *P** |
| --- | --- | --- |
| Adequately Fix Specimens | 2.600  3.800 | .051 |
| Gross Over Cap | 3.800  3.000 | .19 |
| Gross Past 6PM | 3.200  3.600 | .51 |
| Cases Prior to Signout | 2.000  2.800 | .19 |
| Cases on Signout Day | 3.200  3.400 | .74 |
| Adequately Preview | 2.400  3.200 | .19 |
| Review IHC | 2.200  4.000 | .0039 |
| Graduated Responsibility | 1.800  3.400 | .010 |
| Preparedness for Practice | 3.000  4.400 | .023 |

^*^Comparison of agreement from pre- and post- implementation surveys
