# Supplementary material for: A novel 6-day cycle surgical pathology rotation improves resident satisfaction and maintains Accreditation Council for Graduate Medical Education (ACGME) milestone performance
Source: Acad Pathol. 2023 Jun 30;10(3):100088. doi: 10.1016/j.acpath.2023.100088 (PMC10336254; doi:10.1016/j.acpath.2023.100088)
Supplement: Multimedia component 9 [file mmc9.docx]

Supplemental Table 9: CCC data comparing PGY1 paired cohort pre- and post-6 day cycle.

| Internal Metric | Mean | *P* |
| --- | --- | --- |
| PC1 | 2.333  2.500 | .19 |
| PC2 | 2.417  2.400 | .90 |
| PC3 | 2.083  2.200 | .44 |
| PC4 | 2.250  2.300 | .77 |
| PC5 | 2.000  2.300 | .024 |
| MK1 | 2.417  2.500 | .64 |
| MK2 | 2.083  2.400 | .037 |
