# Supplementary material for: A novel 6-day cycle surgical pathology rotation improves resident satisfaction and maintains Accreditation Council for Graduate Medical Education (ACGME) milestone performance
Source: Acad Pathol. 2023 Jun 30;10(3):100088. doi: 10.1016/j.acpath.2023.100088 (PMC10336254; doi:10.1016/j.acpath.2023.100088)
Supplement: Multimedia component 10 [file mmc10.docx]

Supplemental Table 10: CCC data comparing PGY2 paired cohort pre- and post-6 day cycle.

| Internal Metric | Mean | *P* |
| --- | --- | --- |
| PC1 | 2.750  2.714 | .84 |
| PC2 | 2.750  3.701 | .15 |
| PC3 | 2.875  3.071 | .17 |
| PC4 | 2.625  2.929 | .048 |
| PC5 | 3.000  3.000 | 0 |
| MK1 | 3.000  2.929 | .70 |
| MK2 | 3.000  2.571 | .0016 |
