# Supplementary material for: A novel 6-day cycle surgical pathology rotation improves resident satisfaction and maintains Accreditation Council for Graduate Medical Education (ACGME) milestone performance
Source: Acad Pathol. 2023 Jun 30;10(3):100088. doi: 10.1016/j.acpath.2023.100088 (PMC10336254; doi:10.1016/j.acpath.2023.100088)
Supplement: Multimedia component 11 [file mmc11.docx]

Supplemental Table 11: CCC data comparing PGY3 paired cohort pre- and post-6 day cycle.

| Internal Metric | Mean | *P* |
| --- | --- | --- |
| PC1 | 3.600  3.200 | .18 |
| PC2 | 3.700  3.200 | .12 |
| PC3 | 3.900  3.400 | .0078 |
| PC4 | 3.700  3.500 | .14 |
| PC5 | 3.800  3.300 | .02 |
| MK1 | 3.800  3.000 | <.001 |
| MK2 | 3.700  3.400 | .094 |
