# Supplementary material for: A novel 6-day cycle surgical pathology rotation improves resident satisfaction and maintains Accreditation Council for Graduate Medical Education (ACGME) milestone performance
Source: Acad Pathol. 2023 Jun 30;10(3):100088. doi: 10.1016/j.acpath.2023.100088 (PMC10336254; doi:10.1016/j.acpath.2023.100088)
Supplement: Multimedia component 12 [file mmc12.docx]

Supplemental Table 12: CCC data comparing PGY4 paired cohort pre- and post-6 day cycle.

| Internal Metric | Mean | *P* |
| --- | --- | --- |
| PC1 | 4.125  3.800 | .11 |
| PC2 | 4.500  3.900 | .001 |
| PC3 | 4.125  4.000 | .29 |
| PC4 | 4.000  3.900 | .41 |
| PC5 | 4.125  3.900 | .20 |
| MK1 | 4.500  3.800 | .0015 |
| MK2 | 3.500  3.900 | .010 |
